# Protease-activated receptor 2 protects against VEGF inhibitor-induced glomerular endothelial and podocyte injury

**DOI:** 10.1038/s41598-019-39914-8

**Published:** 2019-02-27

**Authors:** Yuji Oe, Tomofumi Fushima, Emiko Sato, Akiyo Sekimoto, Kiyomi Kisu, Hiroshi Sato, Junichi Sugawara, Sadayoshi Ito, Nobuyuki Takahashi

**Affiliations:** 1 Division of Feto-Maternal Medical Science, Department of Community Medical Support, Tohoku Medical Megabank Organization, Tohoku University, Sendai, 980-8574 Japan; 2 Division of Clinical Pharmacology and Therapeutics, Tohoku University Graduate School of Pharmaceutical Sciences & Faculty of Pharmaceutical Sciences, Sendai, 980-8578 Japan; 3 Division of Nephrology, Endocrinology, and Vascular Medicine, Tohoku University Graduate School of Medicine, Sendai, 980-8574 Japan; 4 Research Fellow of Japan Society for the Promotion of Science, Chiyoda-ku, Tokyo 102-0083 Japan

## Abstract

Vascular endothelial growth factor (VEGF) inhibitors cause glomerular injury. We have recently shown that activation of protease-activated receptor 2 (PAR2) by factor Xa exacerbated diabetic kidney disease. However, the role of PAR2 in glomerular injury induced by VEGF blockade is not known. Herein, we investigated the effect of the lack of PAR2 on VEGF inhibitor-induced glomerular injury. Although administering an anti-VEGF antibody by itself did not show renal phenotype in wild type mice, its administration to mice lacking endothelial nitric oxide synthase (eNOS) caused glomerular injury. Different from what we expected, administration of an anti-VEGF antibody in mice lacking PAR2 and eNOS exacerbated albuminuria and reduced the expression levels of CD31, pro-angiogenic VEGF, and angiogenesis-related chemokines in their kidneys. Podocyte injury was also evident in this model of mice lacking PAR2. Our results suggest that PAR2 is protective against VEGF inhibitor-induced glomerular endothelial and podocyte injury.

## Introduction

Vascular endothelial growth factor (VEGF) inhibitors are used in conjunction with chemotherapy to treat several types of cancer. However, kidney glomerular injury, such as thrombotic microangiopathy (TMA), is observed in a subset of patients and can be a cause of treatment discontinuation^1,2^. Some preeclamptic patients develop kidney injury and hypertension caused by soluble fms-like tyrosine kinase 1, a decoy of VEGF that suppresses angiogenesis^3^. Accordingly, there is an increasing interest in exploring novel therapies for VEGF inhibitor-induced kidney injury.

Hypercoagulability is associated with VEGF inhibition. Fibrin deposition is observed within the glomeruli in VEGF inhibitor-induced TMA^1^. Furthermore, coagulation abnormalities are reported in preeclamptic patients treated with a VEGF inhibitor^4,5^. Coagulation factors have a pleiotropic effect through the activation of protease-activated receptors (PARs), a G protein-coupled receptor family^6^. For instance, tissue factor/VIIa complex or factor Xa activates PAR2, which is abundantly expressed in the kidney^6,7^.

Although several studies, including ours, have shown that PAR2 exacerbates glomerular injury in models of diabetic kidney disease (DKD) or glomerulonephritis^7,8^, the role of PAR2 in VEGF inhibitor-induced kidney injury is controversial. Tissue factor and PAR2 exacerbate preeclampsia and kidney injury in models of antiphospholipid syndrome^9,10^. Conversely, PAR2 signaling contributes to endothelial proliferation/migration and increased pro-angiogenic factors^11,12^. Pro-angiogenic roles of PAR2 on limb ischemia and retinal neovascularization were also shown^13–15^. These findings may indicate that PAR2 protects the glomerular endothelium from damage secondary to VEGF inhibition.

Herein, we demonstrated that a lack of PAR2 in VEGF inhibitor-induced glomerular injury model exacerbated albuminuria, and endothelial and podocyte injury, together with reduced angiogenic markers.

## Results

### Role of PAR2 in kidney injury in anti-VEGF antibody-induced glomerular injury

**Figure 1:**
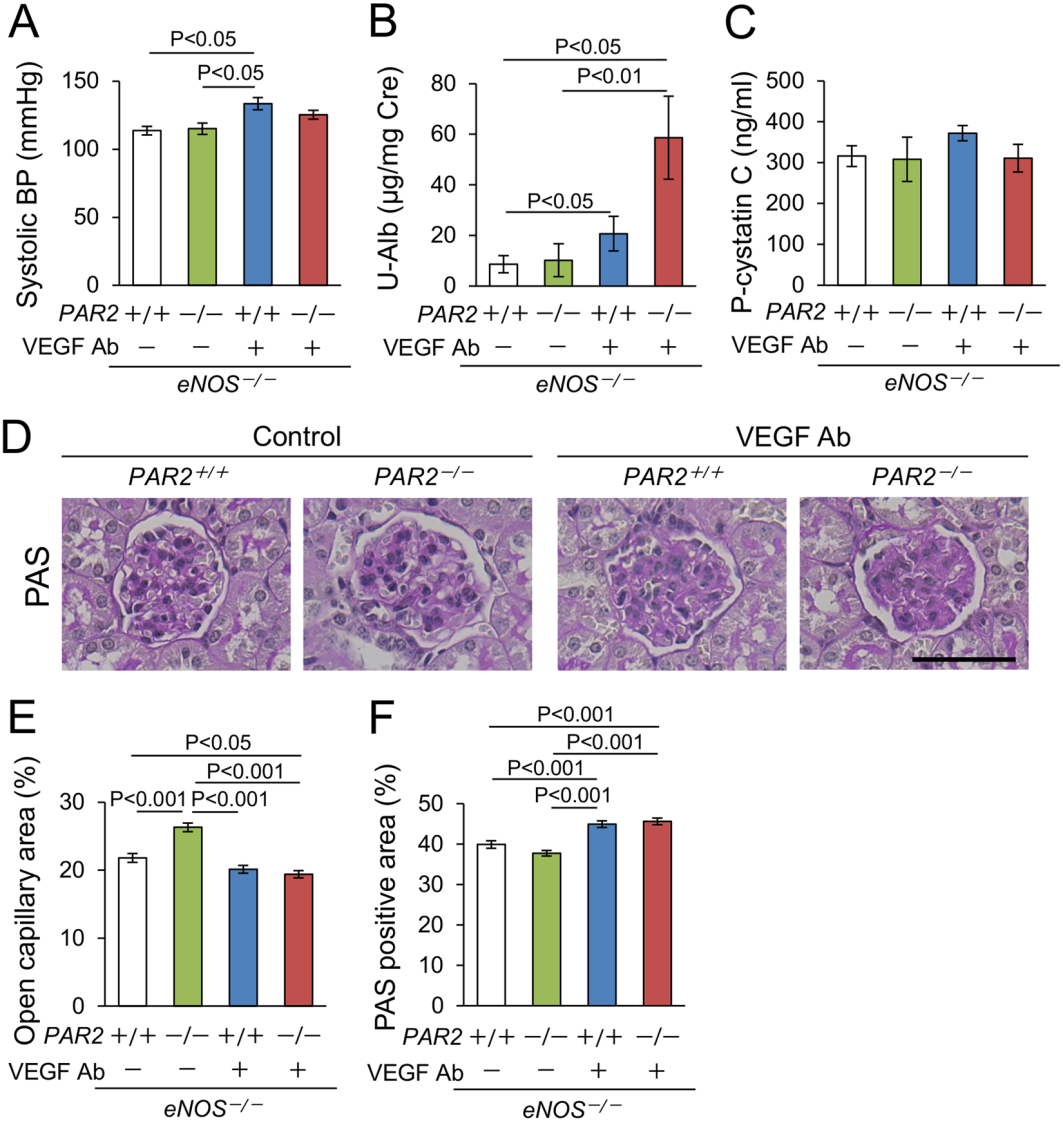
Blood pressure, urinary albumin excretion, and glomerular injury score. (**A**) Systolic blood pressure (BP). (**B**) Urinary albumin excretion (U-Alb). (**C**) Plasma cystatin C (P-cystatin C). (**A**) lack of PAR2 exacerbates albuminuria. n ≥ 5 each group in panel A–C. (**D**) Representative photomicrographs of glomeruli in each group, Periodic acid-Schiff stain (PAS) stain, Scale bar indicates 50 μm. (**E**,**F**) Quantitative data of open capillary and PAS positive area in the glomeruli. Approximately 100 glomeruli each group from more than five mice were evaluated. Cre, creatinine. Ab, antibody. A.U, arbitrary unit. Data are shown as mean ± s.e.m.

To produce a model of mouse kidney injury using an anti-VEGF antibody (Ab), we first tested the effect of anti-VEGF Ab on wild type mice. However, VEGF inhibition did not affect glomerular histology or urinary albumin excretion (Supplementary Fig. 1A,B). Endothelial nitric oxide synthase (eNOS) dysfunction is important in the onset and exacerbation of VEGF inhibitor-induced glomerular injury because eNOS promotes the proliferation and migration of endothelial cells^16^, and because eNOS derived NO is protective against podocyte injury^17^. We have previously shown that a lack of eNOS increases endothelin and exacerbates blood coagulation and preeclampsia^18–20^. Furthermore, eNOS polymorphism is associated with a higher risk of preeclampsia^21^. Accordingly, we next administered an anti-VEGF Ab to *eNOS*^−/−^ mice. To investigate the role of PAR2 in this model, we produced *eNOS*^−/−^ mice with or without PAR2 (*eNOS*^−/−^; *PAR2*^*+/+*^ and *eNOS*^−/−^; *PAR2*^−/−^, respectively), and administered them an anti-VEGF Ab. Blood pressure (BP) levels were increased by VEGF inhibition, whereas PAR2 deletion had no effect (Fig. 1A). A lack of PAR2 significantly increased urinary albumin excretion in *eNOS*^−/−^ mice treated with VEGF inhibitor (58.6 ± 16.4 μg/mg creatinine) compared to that of control groups (Fig. 1B). The level of plasma cystatin C, a marker of renal function, was similar among the groups (Fig. 1C). Two glomerular injury scores (open capillary area and mesangial area) were evaluated as previously demonstrated^7,22^. Open capillary area was increased in *eNOS*^−/−^; *PAR2*^−/−^ mice that did not receive the Ab as compared to that of control *eNOS*^−/−^; *PAR2*^*+/+*^ mice. Anti-VEGF Ab decreased open capillary area in *eNOS*^−/−^; *PAR*^*+/+*^ mice compared to that of *eNOS*^*−/−*^; *PAR2*^*−/−*^ mice that did not receive the Ab and in *eNOS*^−/−^; *PAR2*^−/−^ mice compared to that of the two control groups. Anti-VEGF Ab increased mesangial area compared to that of control groups (Fig. 1D–F). Other basal characteristics were unremarkable (Supplementary table 1). We concluded that in our model, a lack of PAR2 increases urinary albumin excretion and does not protect against renal injury.

### Markers of endothelial cells and podocytes in VEGF inhibitor-treated mice lacking PAR2

**Figure 2:**
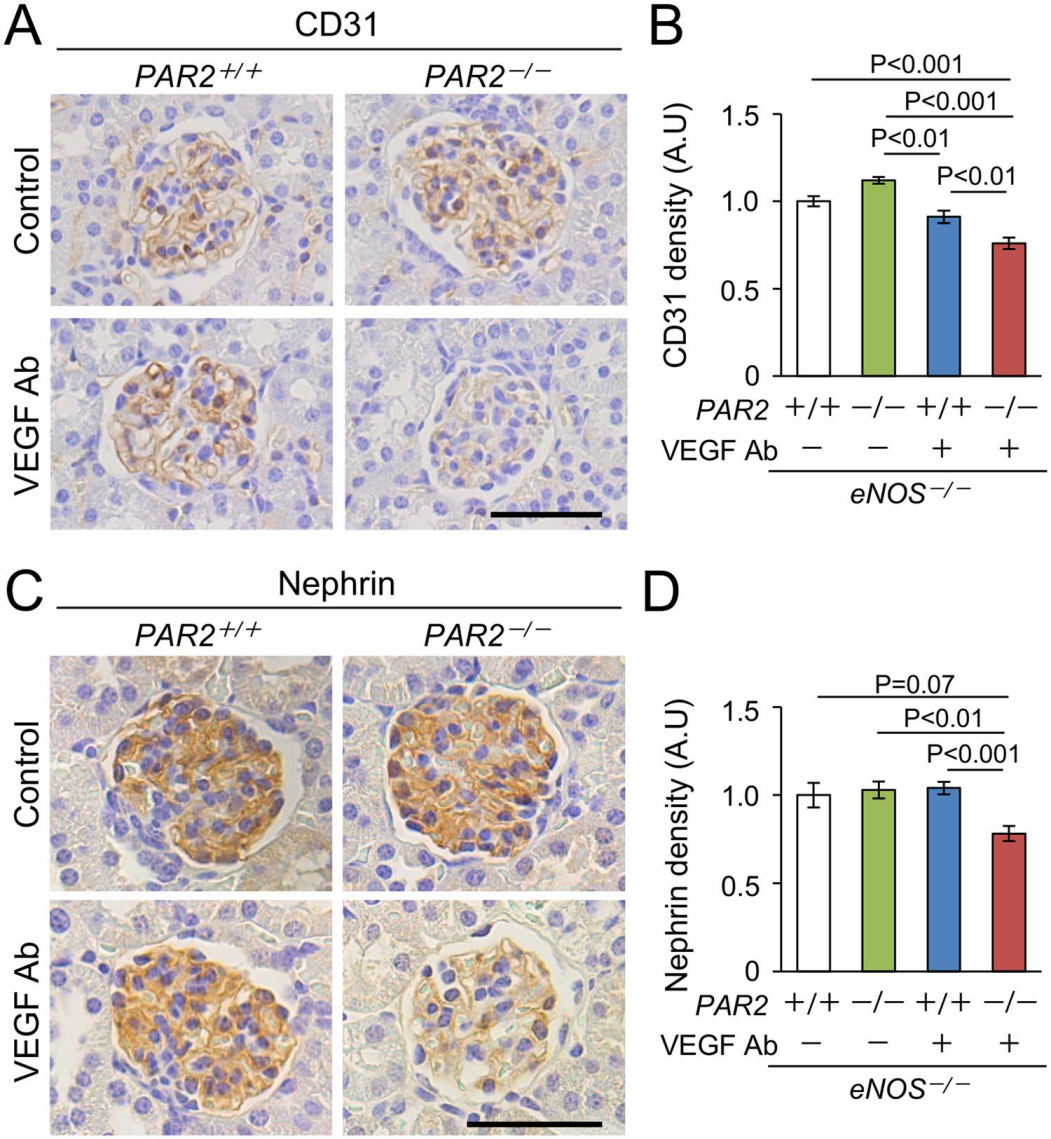
Reduced expression of makers of endothelial cell and podocyte. (**A**) Representative photomicrographs of immunohistochemistry against CD31. Scale bar indicates 50 μm. (**B**) Density of glomerular CD31 is reduced in the kidneys from *eNOS*^*−/−*^; *PAR2*^*−/−*^ with a VEGF inhibitor. (**C**) Representative photomicrographs of immunohistochemistry against nephrin. Scale bar indicates 50 μm. (**D**) Density of glomerular nephrin is reduced in the kidneys from *eNOS*^*−/−*^; *PAR2*^*−/−*^ with a VEGF inhibitor. Approximately 100 glomeruli each group from 4 to 6 mice were evaluated. Ab, antibody. A.U, arbitrary unit. Data are shown as mean ± s.e.m.

Increased urinary albumin excretion could be the result of impaired function of glomerular endothelial cells or podocytes^23,24^. Because VEGF inhibition causes glomerular endotheliosis^1,2^, we first tested whether a lack of PAR2 in the *eNOS*^*−/−*^ mice receiving anti-VEGF Ab damages glomerular endothelial cells. The result showed that a lack of PAR2 reduced glomerular density of immunopositive CD31 (endothelial marker) in the kidneys of the *eNOS*^*−/−*^ mice treated with anti-VEGF Ab (Fig. 2A,B).

Glomerular endothelial cells communicate with podocytes to maintain their function, and glomerular endothelial injury promotes podocyte injury leading to albuminuria^23,24^. Because podocyte dysfunction is known as one of the features of VEGF inhibitor - related glomerular injury^25–27^, we measured nephrin level, which is a podocyte-specific protein. A lack of PAR2 reduced the expression of nephrin in *eNOS*^−/−^ mice receiving anti-VEGF Ab (Fig. 2C,D).

**Figure 3:**
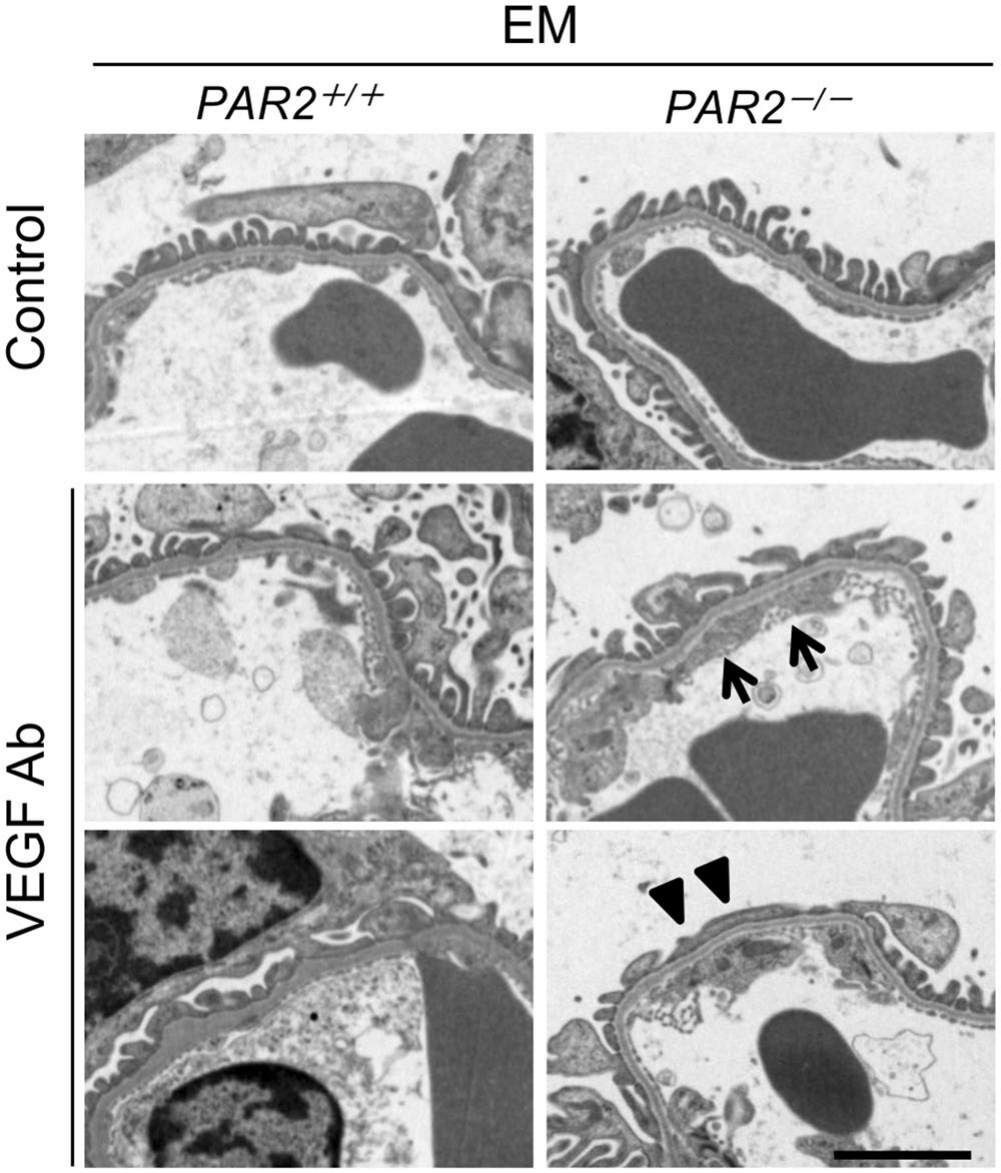
Representative transmission electron micrographs in the glomeruli. Arrows indicate loss of endothelial fenestration. Arrow-heads indicate foot process effacement in podocytes. EM, electron microscopy. Ab, antibody. Scale bar indicates 2 μm.

Consistent with these results, electron microscopy showed a loss of endothelial fenestration and podocyte foot process effacement in the glomeruli from *eNOS*^−/−^*;PAR2*^−/−^ mice treated with anti-VEGF Ab (Fig. 3). We concluded that PAR2 deficiency does not cause damage to endothelial cells or podocytes, but does exacerbate the damage induced by anti-VEGF antibody, which is likely responsible for albuminuria secondary to PAR2 deletion in our model of glomerular injury.

### Expression of angiogenic factors in the kidney

**Figure 4:**
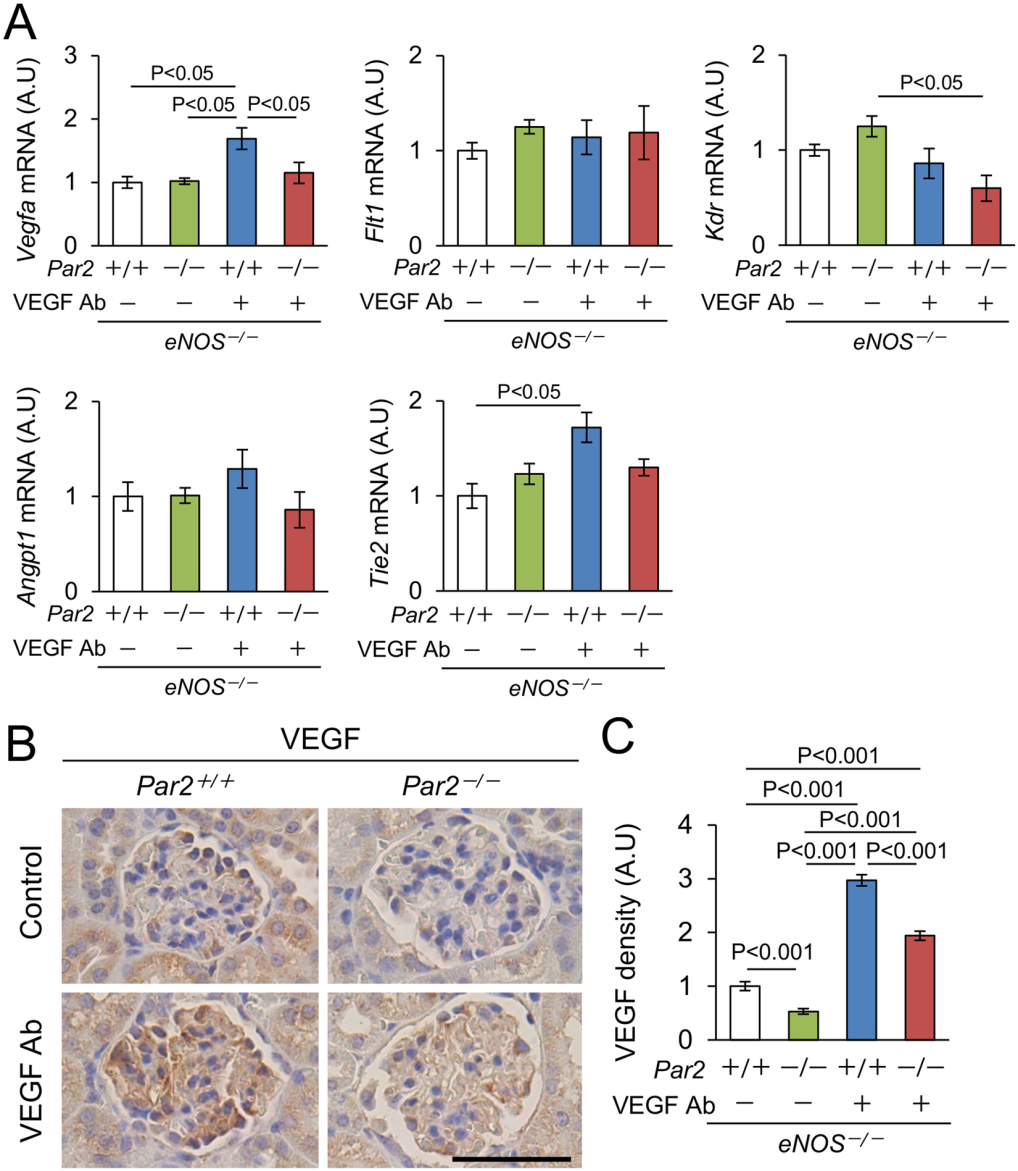
Expression of angiogenic factors in the kidney. (**A**) Gene expression related to pro-angiogenic factors (*Vegfa*, *Angpt1*, *Flt1*, *Kdr*, *and Tie2*). n ≥ 5. (**B**) Representative photomicrographs of immunohistochemistry against VEGF in the kidneys. Scale bar indicates 50 μm. (**C**) Quantitative data of glomerular VEGF protein. Approximately 100 glomeruli each group from 4 to 6 mice were evaluated. Ab, antibody. A.U, arbitrary unit. Data are shown as mean ± s.e.m.

Because PAR2 regulates the expression of angiogenic factors, such as VEGF and angiopoietin^11,15^, we quantified their expression in the kidneys. Among them, the levels of *Vegfa* and *Tie2* were increased in our model, and PAR2 deletion corrected *Vegfa* level (Fig. 4A). Anti-VEGF Ab reduced the expression of *Kdr* in the kidneys from *eNOS*^−/−^; *PAR2*^*−/−*^ mice (Fig. 4A). Consistent with the change in gene expression, the level of glomerular VEGF protein was increased in the kidneys from *eNOS*^−/−^; *PAR2*^*+/+*^ mice treated with anti-VEGF Ab, and a lack of PAR2 reduced it (Fig. 4B,C). Taken together, the exacerbation of glomerular injury by a lack of PAR2 was associated with the reduced expression of angiogenic factors in the kidney.

### Expression of chemokines in the kidney

**Figure 5:**
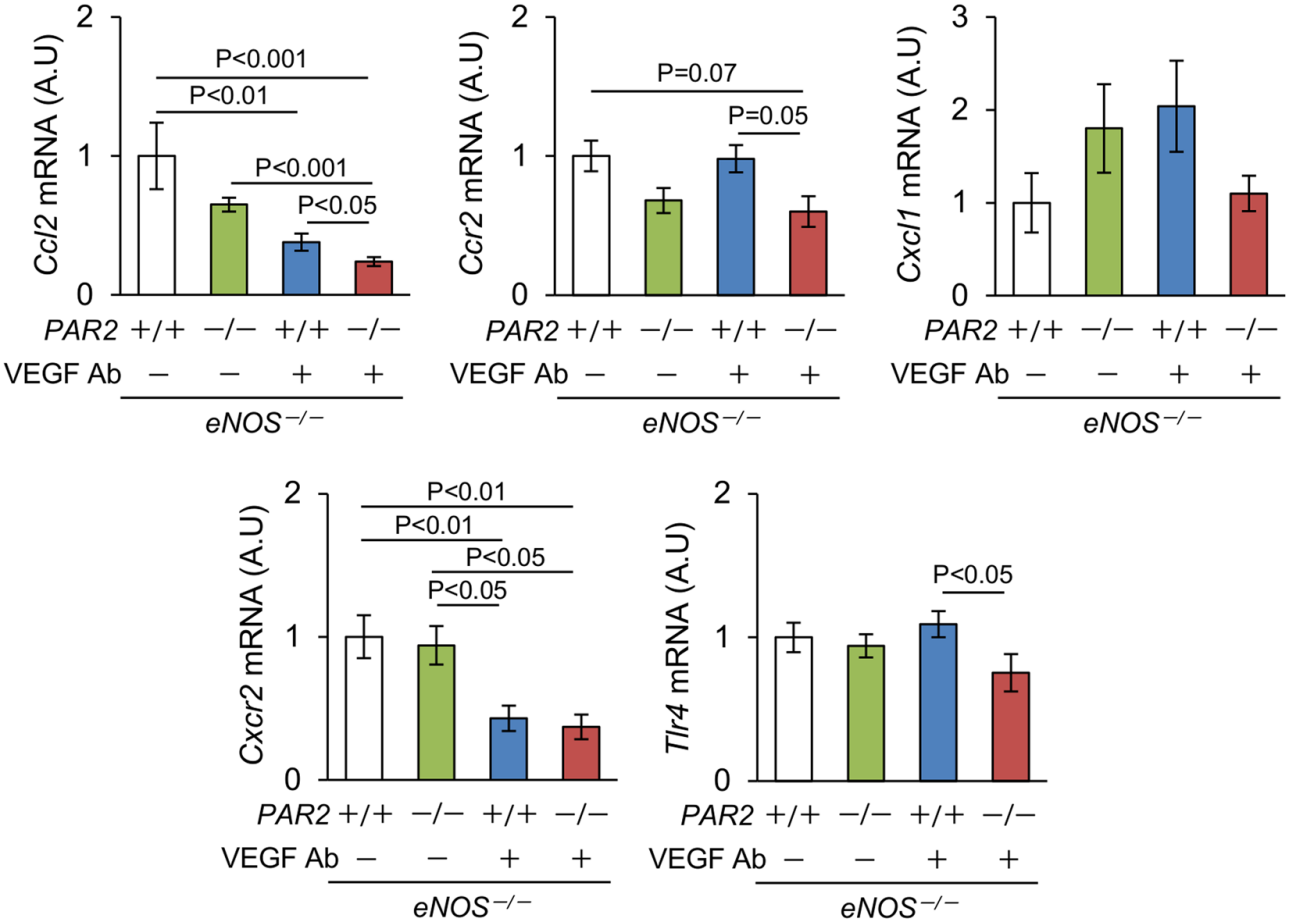
Expression of inflammation - related genes in the kidney. The levels of *Ccl2*, *Ccr2*, *Cxcl1*, *Cxcr2*, and *Tlr4* mRNA in the kidney. n ≥ 5. Ab, antibody. A.U, arbitrary unit. Data are shown as mean ± s.e.m.

Next, we quantified the levels of angiogenesis-related chemokines (Fig. 5). Anti-VEGF Ab decreased *Ccl2* mRNA expression in *eNOS*^−/−^; *PAR2*^*+/+*^ mice and there was a greater decrease in *eNOS*^−/−^; *PAR2*^*−/−*^ mice. The level of *Cxcl1* mRNA was similar among the groups. Non-significant reduction of *Ccr2* expression was obtained by a lack of PAR2 in the presence and absence of anti-VEGF Ab. The level of *Cxcr2* was not affected by the PAR2 genotype, but was reduced in the kidneys from mice receiving anti-VEGF Ab. TLR4 signaling is known to regulate angiogenesis^28^, and a lack of PAR2 significantly reduced the level of *Tlr4* in the kidneys from *eNOS*^−/−^ mice receiving anti-VEGF Ab. As opposed to the changes in inflammatory genes, the number of infiltrated MAC2 positive cells, a marker of macrophages, was similar among the groups (Supplementary Fig. 2).

### Effect of PAR2 agonist on human endothelial cells

**Figure 6:**
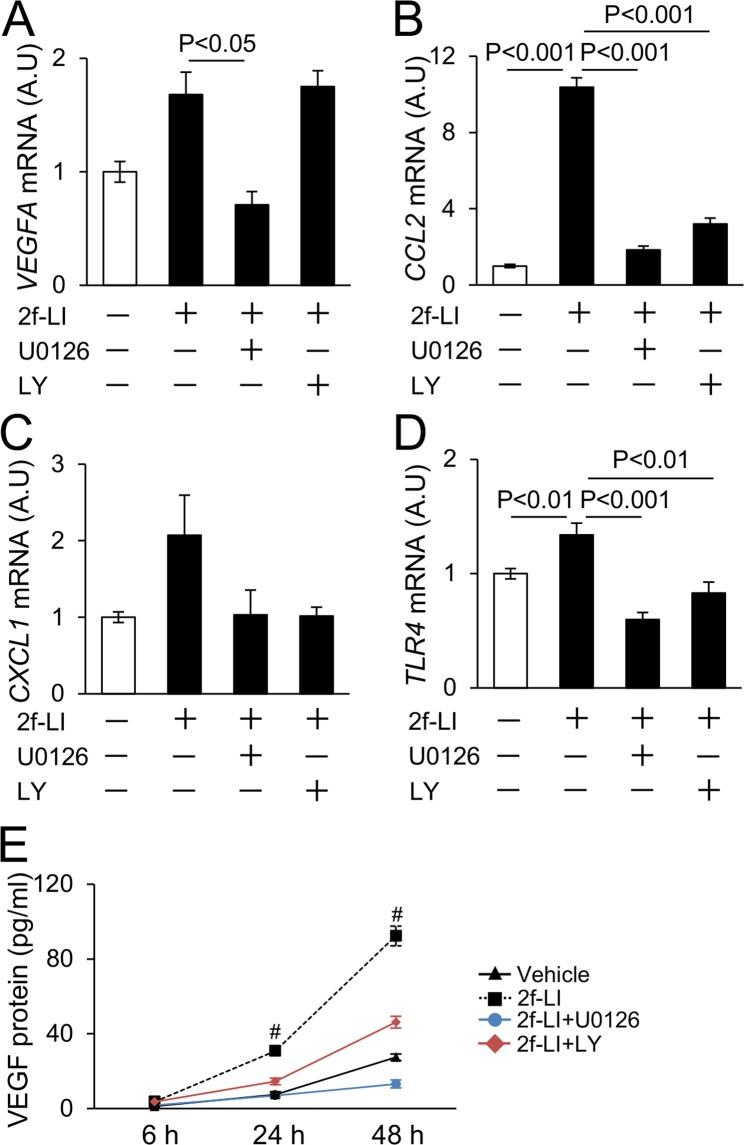
Expression of angiogenesis - related genes in human endothelial cell treated with PAR2 agonist. (**A**–**D**) Effect of PAR2 agonist (2f-LIGRLO, 20 μM), MAPK inhibitor (U0126, 10 μM), and PI3K inhibitor (LY294002, 10 μM) on expression of *VEGFA* and inflammatory-related genes. Increased expression of *VEGFA* is reduced by U0126. Expression levels of *CCL2*, *CXCL1*, and *TLR4* are reduced by both U0126 and LY294002. Cells were treated for 3 hrs. (**E**) The protein level of VEGF in conditioned media harvested from cultured endothelial cells. 2f-LIGRLO increases VEGF protein after 24 or 48 hrs treatment. Both U0126 and LY294002 reduce it. ^#^P < 0.01 vs Vehicle, 2f-LI + U0126, and 2f-LI + LY groups. A.U, arbitrary unit. 2f-LI, 2f-LIGRLO. LY, LY294002. Experiments were repeated 3 times. Data are shown as mean ± s.e.m.

Because endothelial cells closely interact with coagulation factors and highly express PAR2^6^, we tested the direct effect of the PAR2 agonist, 2f-LIGRLO, on pro-angiogenic factors using a human endothelial cell line (EA.hy926). After treatment of EA.hy926 cells with 2f-LIGRLO (20 μM) for 3 hours, the expression levels of *VEGFA*, *CCL2*, *CXCL1*, and *TLR4* mRNA were significantly elevated (Supplementary Fig. 3). Because PAR2 signaling is associated with ERK and PI3K/Akt signaling^6^, we next tested the effect of MAPK inhibitor (U0126) and PI3K inhibitor (LY294002) on these changes. U0126 significantly reduced elevated *VEGFA* expression by 2f-LI, whereas LY294002 did not. Elevated *CCL2*, *CXCL1*, and *TLR4* mRNA were reduced by both U0126 and LY294002 treatments (Fig. 6A–D). The level of VEGF protein in conditioned media harvested from cultured cells was assessed using ELISA. 2f-LIGRLO increased VEGF protein after 24 or 48 hours of treatment with the agonist that was reduced by U0126. On contrary to the changes of mRNA, LY 294002 partially inhibited VEGF production (Fig. 6E). These results show that the expression of VEGF, chemokines, and TLR4 by PAR2 agonist is regulated by both MAPK and PI3K.

### Tissue factor expression and fibrin/fibrinogen deposition in the kidney

We next characterized the coagulation abnormalities in our model. Fibrin/fibrinogen thrombi and increased immunoreactive tissue factor were not observed in the glomeruli from eNOS wild type mice regardless of treatment with anti-VEGF Ab or PAR2 expression (Supplementary Fig. 4A). Conversely, glomeruli in which fibrin/fibrinogen thrombi were deposited were easily observed in the kidney from anti-VEGF Ab-treated mice lacking eNOS (Supplementary Fig. 4B). Furthermore, anti-VEGF Ab increased the expression level of tissue factor in mice lacking eNOS, whereas lack of PAR2 did not affect it (Supplementary Fig. 4B,C).

## Discussion

We have previously shown that PAR2 exacerbates DKD and adenine-induced chronic kidney disease^7,29^. Based on these findings, we postulated that a lack of PAR2 would alleviate VEGF inhibitor-induced glomerular injury. However, contrary to our postulation, our results from the present study show that a lack of PAR2 in the *eNOS*^−/−^ mice receiving anti-VEGF Ab worsens kidney injury (albuminuria) and endothelial and podocyte injury.

Increased urinary albumin excretion secondary to a lack of PAR2 in *eNOS*^−/−^ mice receiving anti-VEGF Ab is likely caused by endothelial and podocyte injury, which impairs the filtration barrier^23^. The decreased expression of CD31 and nephrin, and the dysfunction of endothelial cells and podocytes in our model suggest that the damage to the endothelial cells and podocytes is likely responsible for albuminuria.

Our finding that healthy endothelial cells cannot be maintained in the lack of PAR2 suggests that it plays a pivotal role in glomerular endothelial protection. Indeed, it was previously reported in the literature that PAR2 directly promotes the expression of angiogenic factors such as VEGF, angiopoietins, and their receptors in several cell lines^11,15,30^. PAR2 activation promotes endothelial proliferation in primary neuroretinal endothelial cells via pro-inflammatory effect^11^. PAR2 is indispensable for tissue factor-induced microvessel stabilization^12^. Moreover, PAR2 is essential in retinal angiogenesis in rodent models^13,15^. These findings are consistent with our observations. We therefore investigated the protective role of PAR2 in glomerular endothelial injury caused by VEGF inhibition, and several pathways above cooperatively contribute to reno-protection in our model.

PAR2 is known to increase cytokine/chemokine expression^6^. CCL2–CCR2 and CXCL1–CXCR2 pathways, well-described pro-angiogenic chemokines, mediate corneal neovascularization, hepatic angiogenesis, endothelial recovery in arterial injury, and cancer-related angiogenesis^31–36^. Furthermore, previous reports have shown that TLR4, another inflammatory mediator, contributes to angiogenesis; TLR4 deletion reduced angiogenesis in alkali-induced corneal neovascularization, ischemic neural tissue, and hindlimb ischemia^37–39^. Our experiment using human endothelial cell line demonstrated that PAR2 agonist increased the level of *TLR4* expression. Similarly, PAR2 deletion reduced the expression of *TLR4* in the kidneys from VEGF inhibitor-treated mice. Although how angiogenesis-related chemokines contribute to repair of glomerular endothelial cells under VEGF inhibition requires further examination, since such inflammatory mediators are known to promote endothelial survival or production of other angiogenic factors^32^ which were likely protective in our model of glomerular injury.

The main source of glomerular VEGF is podocytes^1^, however, PAR2 agonist did not increase VEGFA in immortalized murine podocytes in our experiment (data not shown). Interestingly, we found that PAR2 increases production of VEGF in endothelial cells. Based on the literature, endothelial VEGF protects itself by autocrine/paracrine manner^40,41^, which supports the protective role of PAR2 in our model. A lack of PAR2 did not affect glomerular macrophage infiltration, another source of VEGF^42^, in anti-VEGF Ab-treated mice. A limitation of this study is that we used systemic knock-out of PAR2. Further studies are required to elucidate how PAR2 is involved in angiogenesis by podocytes, endothelial cells, and macrophages using conditional knock-out of PAR2, which is our future plan.

Podocyte damage is common and an important finding of VEGF inhibitor-induced kidney injury; urinary podocyte excretion is increased in patients treated with bevacizumab or in those with preeclampsia^26,27^. Endothelial injury is known to promote podocyte injury. Previous papers have shown that endothelial oxidative stress and reactive oxygen species generation is associated with podocyte detachment in a focal segmental sclerosis model^23^. Other study has demonstrated that exosomes derived from endothelial cells treated with high glucose exacerbate podocyte dysfunction^43^. Collectively, increased endothelial injury by a lack of PAR2 likely causes secondary podocyte injury, which is likely important in our model of glomerular injury. Although VEGF signaling in podocyte is still controversial^44,45^, a previous report has shown that VEGFR2 interacts with nephrin, a specific podocyte marker^45^. How VEGF inhibitor and/or PAR2 directly affect podocyte maintenance and function is still unclear and should be investigated in the future.

A lack of PAR2 worsens glomerular injury in mice lacking eNOS treated with VEGF inhibitor, but not in eNOS wild type mice treated with VEGF inhibitor. It is likely that the lack of eNOS up-regulates coagulation in models of kidney injury^19,20^, increases TF, and activates coagulation cascade and PAR2. We have shown that VEGF inhibition increased glomerular TF expression in mice lacking eNOS. This finding suggests that a combination of eNOS deficiency and VEGF inhibition may up-regulate TF-PAR2 pathway which has a protective role in VEGF inhibitor-induced glomerular injury.

Both DKD and VEGF inhibitor-induced glomerulopathies are hypercoagulable states. A lack of PAR2 decreases VEGF and pro-angiogenic cytokines in both DKD and VEGF inhibitor-induced glomerular injury (ref.^7^ and our preliminary observation). However, the lack or inhibition of PAR2 ameliorates DKD^7,46^, whereas it worsens VEGF inhibitor-induced glomerular injury. DKD is characterized by abnormally high VEGF expression and enhanced angiogenesis^47,48^. Furthermore, blockade of VEGF signaling ameliorates DKD in rodent models^49^. The inhibition of factor Xa, which suppresses the activation of PAR2, alleviates DKD^7,50^. Collectively, these findings indicate that the coagulation and activation of PAR2 promote excessive VEGF production and abnormal angiogenesis, and are pathogenic in DKD. On the contrary, glomerular endothelial injury in our current model is caused by inhibiting the effect of VEGF by anti-VEGF Ab. In this setting, PAR2-driven increase in the production of VEGF and angiogenesis-related chemokines probably maintain healthy glomerular endothelium. The contrasting effect of PAR2 on DKD and anti-VEGF model is consistent with the previous findings that both too high and too low levels of VEGF are pathogenic, and that the window of VEGF level needed to maintain healthy glomeruli/endothelial cells is very narrow^51^.

Although VEGF inhibition caused glomerular endothelial injury, features of platelet activation (glomerular platelet deposition or thrombocytopenia) and hemolytic anemia were unremarkable (data not shown). Furthermore, acute kidney failure that is common in human TMA^1,2^ was lacking. There was no evidence for the involvement of TMA in our model. VEGF inhibitors cause another form of glomerular injury, minimal change glomerulopathy/focal segmental glomerulopathy, which is characterized by proteinuria with prominent podocyte injury, increased c-mip, and less inflammation^52,53^. Our model could explain the pathogenesis of renal injury in these patients.

In conclusion, we found that PAR2 promotes pro-angiogenic action and is reno-protective in VEGF inhibitor-induced kidney injury.

## Methods

### Animal model

All experiments were conducted in compliance with the guidelines of Tohoku University. The Institutional Animal Care and Use Committee at Tohoku University approved the experimental protocol. Ten to fourteen-week-old female *eNOS*^+/+^; *PAR2*^+/+^, *eNOS*^+/+^; *PAR2*^−/−^, *eNOS*^−/−^; *PAR2*^+/+^, or *eNOS*^−/−^; *PAR2*^−/−^ mice with C57BL/6J genetic background^7^ were used. *eNOS*^−/−^ or *eNOS*^−/−^; *PAR2*^−/−^ mice were obtained by mating male and female *eNOS*^+/−^; *PAR2*^+/−^ mice. Thereafter, *eNOS*^−/−^ or *eNOS*^−/−^; *PAR2*^−/−^ littermate colony was individually maintained. These mice were injected with B20–4.1.1 (5 mg/kg), a mouse anti-VEGF Ab, on day 0 and 4^54^. The samples were collected on day 7. B20-4.1.1 was kindly provided by Genentech Inc. for research use (South San Francisco, CA, USA). Control groups received a vehicle. Our preliminary observations demonstrated that IgG isotype does not show apparent kidney injury in our experimental condition (data not shown).

### Biochemical measurement

ELISA kits were used to measure plasma cystatin C (R&D Systems, Inc., Minneapolis, MN) and human VEGF protein in conditioned media harvested from cultured cells (R&D Systems, Inc., Minneapolis, MN) according to the manufacturer’s protocol.

### Urinary analysis

Spot urine samples were collected on day 7. ELISA kit was used to measure urinary albumin (Exocell Inc., Philadelphia, PA). Urinary creatinine was determined by the method we developed using LC-MS/MS^55^. Urinary albumin to creatinine ratio was defined as urinary albumin excretion.

### BP measurement

BP was measured by the computerized tail-cuff method using CODA system (Kent Scientific Corporation, Torrington, CT) on day 6. All procedures were performed as previously described^7,22^.

### Real-time quantitative PCR

Total RNA from the kidney was extracted using TRI Reagent (Molecular Research Center, Inc., Cincinnati, OH). Reverse transcription reaction and real-time PCR were performed using iScript Advanced cDNA Synthesis kit and SsoAdvanced Universal Probe/SYBR Supermix kit (Biorad, Hercules, CA) according to the manufacturer’s protocol. Hypoxanthine-guanine phosphoribosyltransferase (*Hprt*) was used as a reference gene as we previously reported^7,29^. The sequences of primers are available on request.

### Histological evaluation

Fixed kidney samples were embedded in paraffin, and sections 1.5 μm in thickness were stained with Periodic acid-Schiff (PAS) stain. Glomeruli with a similar diameter of maximal size containing vascular pole were randomly chosen, so that the glomeruli were all approximately axial. The glomerular open capillary area was expressed as the ratio of the glomerular tuft area. The mesangial matrix score was defined as the ratio of glomerular PAS positive area to glomerular tuft area. All examinations were quantified using ImageJ (National Institute of Health, Bethesda, MD) as we previously described^7,18,22^.

### Immunohistochemistry

For immunohistochemistry, rat anti-mouse CD31 antibody (0.3125 μg/ml, BD Pharmingen, Franklin Lakes, NJ), goat anti-mouse VEGF antibody (0.33 μg/ml, R&D Systems, Inc., Minneapolis, MN), goat anti-human nephrin antibody (0.4 μg/ml, Santa Cruz Biotechnology, Dallas, TX), goat anti-mouse tissue factor (4 μg/ml, R&D Systems, Inc., Minneapolis, MN), rabbit anti-human fibrin/fibrinogen antibody (1.8 μg/ml, Dako, Denmark), and rat anti-human/mouse galectin3 (MAC2) antibody (0.5 μg/ml, ebioscience, San Diego, CA) were used. Heat–induced antigen retrieval was performed using sodium citrate buffer to detect VEGF, fibrin, and MAC2. Proteinase K (Dako, Denmark) was used to detect CD31, nephrin, and tissue factor. Primary antibodies were incubated overnight at 4 °C. *N*-Histofine simple stain kits (Nichirei biosciences Inc., Tokyo, Japan) were used as a secondary antibody according to the manufacturer’s protocol. We incubated sections with IgG isotype or without primary antibody as a negative control. Glomerular density of each protein was assessed using Image J (National Institute of Health, Bethesda, MD).

### Culture of human endothelial cells

Human endothelial cells (EA.hy926) were cultured in DMEM-H containing 10% fetal bovine serum^56^. 2f-LIGRLO was purchased from Tocris Bioscience (Bristol, United Kingdom). U0126 was obtained from Wako Pure Chemical Industries (Osaka, Japan). LY294002 was obtained from Sigma (St. Louis, MO). All experiments were performed after serum starvation for 24 hrs. Both U1026 and LY294002 were administered an hour before 2f-LIGRLO treatment. For quantitative PCR analysis, cells were harvested after 3 hrs of incubation with 2f-LIGRLO. For protein analysis, conditioned media were harvested from cultured endothelial cells after 6, 24, and 48 hrs of incubation with 2f-LIGRLO.

### Statistics analysis

Multiple groups were compared using two-way ANOVA with the Tukey-Kramer test for parametric values after checking normality and equal variance. If not pass the tests, log transformation was applied or Kruscal-Wallis test followed by Steel-Dwass test was used for non-parametric values. All analyses were performed using JMP 11.0.0 (SAS Institute Inc., Cary, NC). Values are presented as mean ± s.e.m. Differences were considered statistically significant with P < 0.05.

## Supplementary information

Supplementary information
