# Supplementary material for: Protease-activated receptor 2 protects against VEGF inhibitor-induced glomerular endothelial and podocyte injury
Source: Sci Rep. 2019 Feb 27;9:2986. doi: 10.1038/s41598-019-39914-8 (PMC6393426; doi:10.1038/s41598-019-39914-8)
Supplement: Supplementary file 1 — Supplementary information [file 41598_2019_39914_MOESM1_ESM.pdf]

Supplementary information

Protease-activated receptor 2 protects against VEGF inhibitor – induced glomerular endothelial and podocyte injury

Yuji Oe[1,4], Tomofumi Fushima[2], Emiko Sato[2,3], Akiyo Sekimoto[2,3], Kiyomi Kisu[3], Hiroshi Sato[2,3], Junichi Sugawara[1], Sadayoshi Ito[3], and Nobuyuki Takahashi[2,3]

[1] Division of Feto-Maternal Medical Science, Department of Community Medical Support, Tohoku Medical Megabank Organization, Tohoku University, Sendai 980-8574, Japan.

[2] Division of Clinical Pharmacology and Therapeutics, Tohoku University Graduate School of Pharmaceutical Sciences & Faculty of Pharmaceutical Sciences, Sendai 980-8578, Japan.

[3] Division of Nephrology, Endocrinology, and Vascular Medicine, Tohoku University Graduate School of Medicine, Sendai 980-8574, Japan.

[4] Research Fellow of Japan Society for the Promotion of Science, Chiyoda-ku, Tokyo 102-0083, Japan.

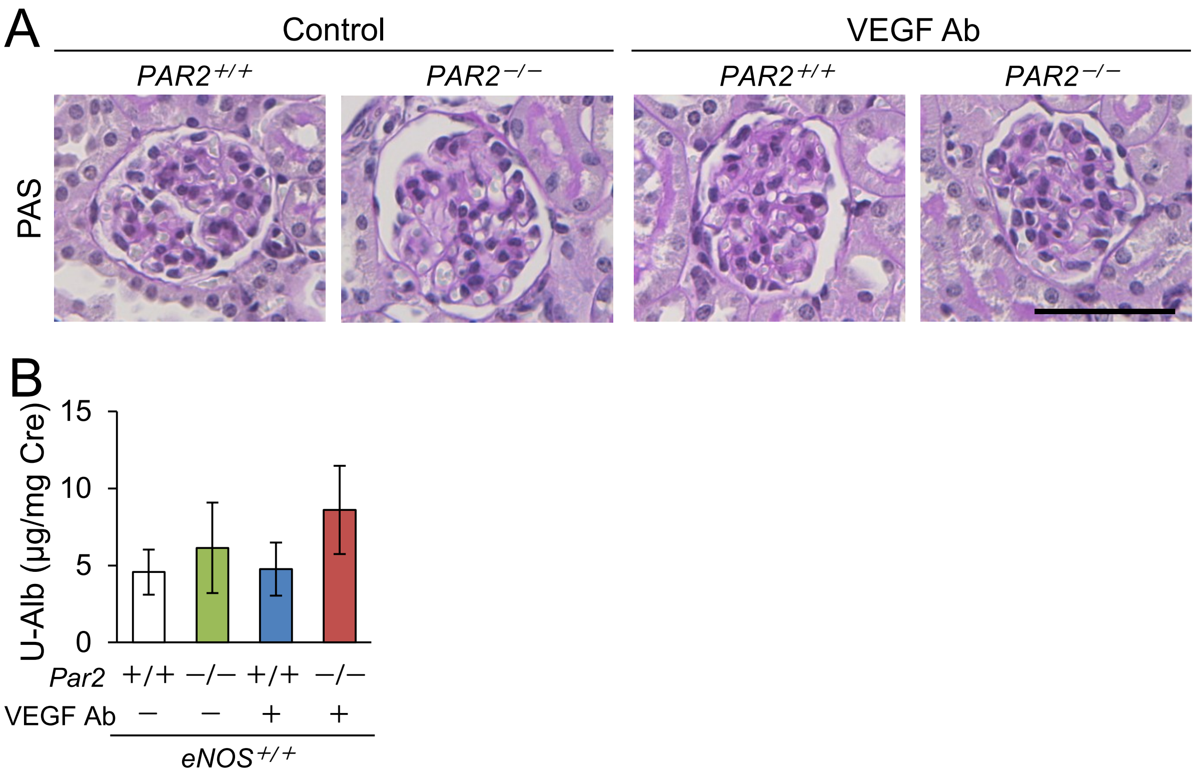

Supplementary figure 1. Effect of VEGF inhibition on *eNOS*$^{+/+}$ mice

A. Representative photomicrographs of PAS stain. Apparent glomerular change is not observed in PAS stain. Scale bar indicates 50 μm. **B.** The level of urinary albumin excretion (U-Alb) is similar among the group. Cre, creatinine. Ab, antibody. A.U, arbitrary unit. n≥5. Data are shown as mean ± s.e.m.

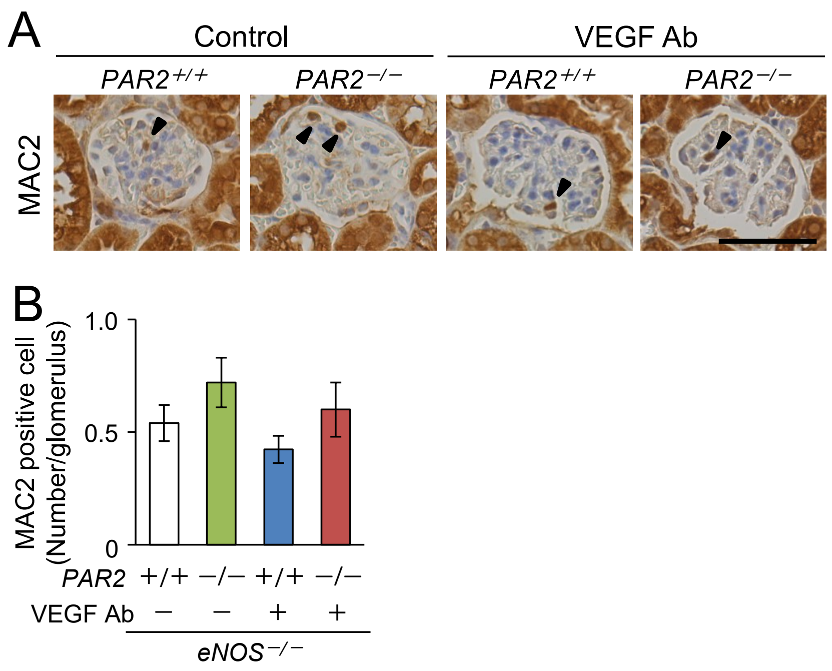

Supplementary figure 2. MAC2 positive cells in the kidney

A. Representative photomicrographs of glomerular MAC2 stain. Scale bar indicates 50 μm. **B.** The number of MAC2 positive cells in the glomeruli. Approximately 100 glomeruli each group from 4 to 6 mice were counted and compared. Ab, antibody. A.U, arbitrary unit. Data are shown as mean ± s.e.m.

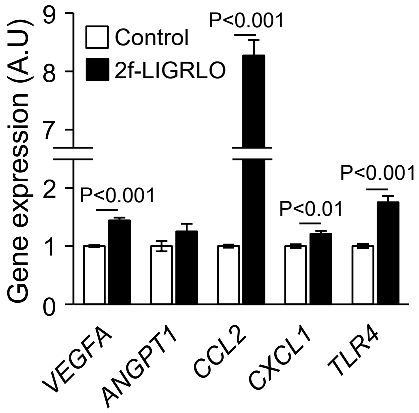

Supplementary figure 3. Effect of PAR2 agonist on human endothelial cells

PAR2 agonist, 2f-LIGRLO (20 μM), increases the expression levels of *VEGFA*, *CCL2*, *CXCL1*, and *TLR4* in human endothelial cell (EA. hy 926). That of *ANGPT1* is unchanged. Cells were treated for 3 hrs. Experiments were repeated 3 times. Data are shown as mean ± s.e.m.

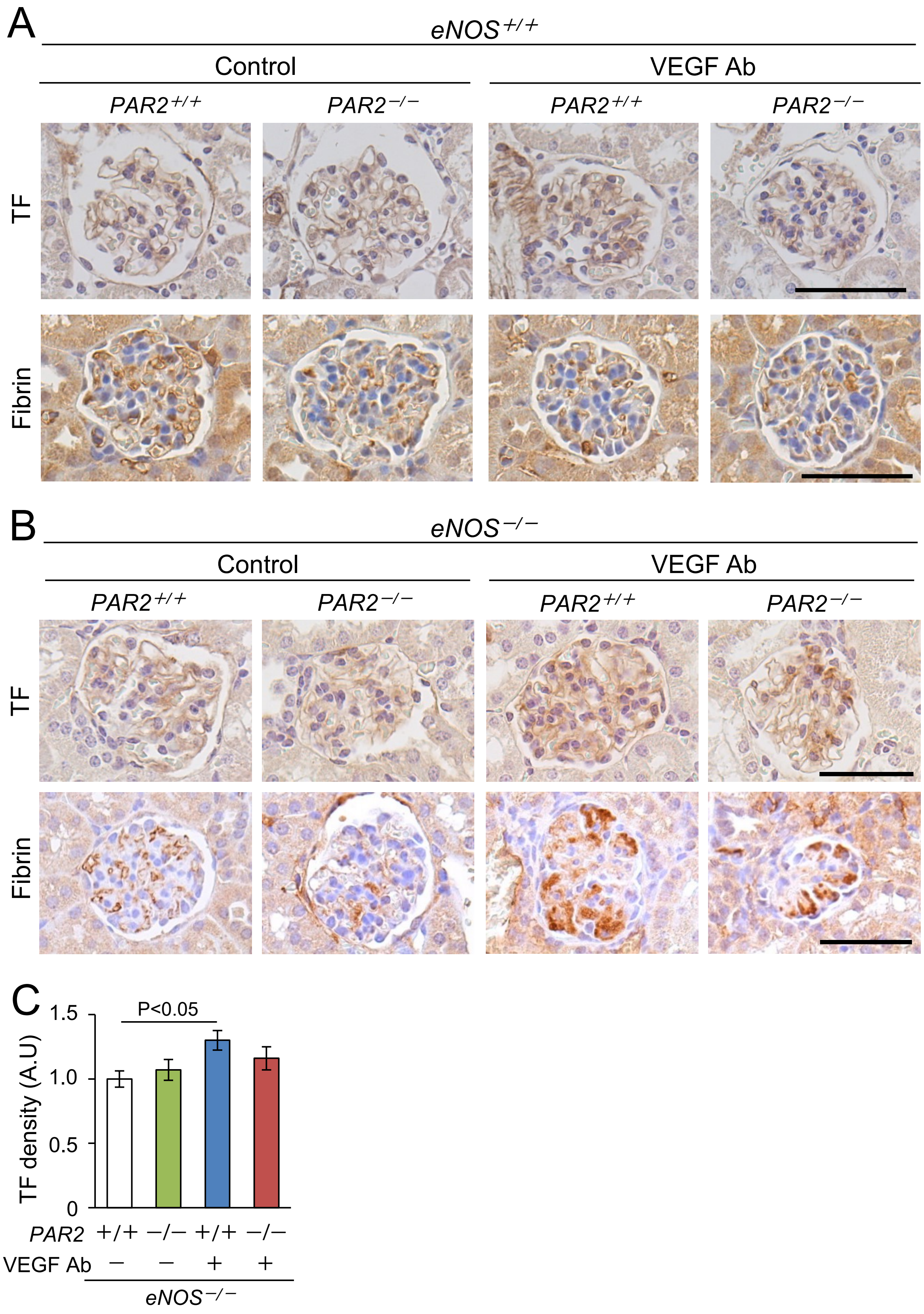

Supplementary figure 4. A. Representative photomicrographs of immunohistochemistry against tissue factor (TF) and fibrin/fibrinogen in the kidney from eNOS wild-type mice. Scale bar indicates 50 μm. **B.**

Representative photomicrographs of immunohistochemistry against fibrin/fibrinogen and TF in the kidney from eNOS null mice. Deposited fibrin/fibrinogen thrombi are observed in the glomeruli from anti-VEGF Ab treated mice. Scale bar indicates 50 μm. **C**. Quantitative data of glomerular TF protein. Approximately 100 glomeruli each group from 4 to 6 mice were evaluated. Ab, antibody. A.U, arbitrary unit. Data are shown as mean ± s.e.m.

Supplementary Table 1. Basal characteristics of studied mice

	eNOS$^{-/-}$; *PAR2*$^{+/+}$ Vehicle	*eNOS*$^{-/-}$; *PAR2*$^{-/-}$ Vehicle	*eNOS*$^{-/-}$; *PAR2*$^{+/+}$ Anti-VEGF Ab	*eNOS*$^{-/-}$; *PAR2*$^{-/-}$ Anti-VEGF Ab
BW (g)	19.3 ± 0.5	19.4 ± 0.3	20.3 ± 0.5	20.3 ± 0.3
Systolic BP (mmHg)	113.8 ± 3.2	115.1 ± 4.1	133.5 ± 4.5[ab]	125.4 ± 3.3
Diastolic BP (mmHg)	82.5 ± 4.9	88.4 ± 3.2	102.7 ± 4.5[a]	95.3 ± 2.8
Rt kidney wt (mg)	86.9 ± 6.1	89.3 ± 5.2	89.8 ± 5.1	102.9 ± 3.6
Rt kidney/BW (mg/g)	4.5 ± 0.3	4.6 ± 0.3	4.5 ± 0.3	5.1 ± 0.2
Liver wt (mg)	889.1 ± 28.3	849.8 ± 26.0	870.7 ± 32.9	868.0 ± 23.6
Liver wt/BW (mg/g)	46.0 ± 1.0	43.9 ± 1.0	42.9 ± 1.0	42.9 ± 1.2
Plasma cystatin C (ng/ml)	316.0 ± 25.3	307.8 ± 54.3	371.9 ± 18.8	310.5 ± 33.9
Urinary Alb (μg/mg Cre)	8.7 ± 3.5	10.2 ± 6.5	20.7 ± 6.8[a]	58.6 ± 16.4[ac]

Data are mean ± s.e.m. n ≥ 6. Abbreviations: BW, body weight; BP, blood pressure; Rt, right; wt, weight; Alb, albumin; Cre, creatinine; Ab, antibody

a, P<0.05 vs *eNOS*$^{-/-}$; *PAR2*$^{+/+}$ b, P<0.05 vs *eNOS*$^{-/-}$; *PAR2*$^{-/-}$ c, P<0.01 vs *eNOS*$^{-/-}$; *PAR2*$^{-/-}$
